# Downregulation of dystroglycan glycosyltransferases LARGE2 and ISPD associate with increased mortality in clear cell renal cell carcinoma

**DOI:** 10.1186/s12943-015-0416-z

**Published:** 2015-07-30

**Authors:** Michael R. Miller, Deqin Ma, James Schappet, Patrick Breheny, Sarah L. Mott, Nadine Bannick, Eric Askeland, James Brown, Michael D. Henry

**Affiliations:** Department of Molecular Physiology and Biophysics, University of Iowa Carver College of Medicine, 6-510 Bowen Science Bldg, Iowa, USA; Department of Pathology, University of Iowa Carver College of Medicine, Iowa, USA; Institute for Clinical and Translational Sciences, Iowa, USA; Department of Biostatistics, University of Iowa Carver College of Medicine, Iowa, USA; Holden Comprehensive Cancer Center, Iowa, USA; Department of Urology, University of Iowa Carver College of Medicine, Iowa, USA

**Keywords:** (MESH Terms), Dystroglycans, Carcinoma, Renal cell, Glycosylation

## Abstract

**Background:**

Dystroglycan (DG) is a cell-surface laminin receptor that links the cytoskeleton to the extracellular matrix in a variety of epithelial tissues. Its function as a matrix receptor requires extensive glycosylation of its extracellular subunit αDG, which involves at least 13 distinct genes. Prior work has shown loss of αDG glycosylation in an assortment of carcinomas, including clear cell renal cell carcinoma (ccRCC) though the cause (s) and functional consequences of this loss are still unclear.

**Methods:**

Using The Cancer Genome Atlas (TCGA) database, we analyzed the DG glycosylation pathway to identify changes in mRNA expression and correlation with clinical outcomes. We validated our findings with a cohort of 65 patients treated with radical nephrectomy by analyzing DG glycosylation via immunohistochemistry and gene expression via qRT-PCR.

**Results:**

Analysis of TCGA database revealed frequent dysregulation of a subset of DG glycosyltransferases. Most notably, there was a frequent, significant downregulation of *GYLTL1B* (LARGE2) and *ISPD*. DG glycosylation is frequently impaired in ccRCC patient samples and most strongly associates with downregulation of *GYLTL1B.*

**Conclusions:**

Reduced levels of *GYLTL1B* and *ISPD* mRNA associated with increased patient mortality and are the likely cause of αDG hypoglycosylation in ccRCC.

**Electronic supplementary material:**

The online version of this article (doi:10.1186/s12943-015-0416-z) contains supplementary material, which is available to authorized users.

## Introduction

Renal cell carcinoma is a highly prevalent disease that will newly affect approximately 64,000 people in 2014 [1]. ccRCC is the most common histologic subtype of renal cell carcinoma and exhibits a 5-year disease-specific survival rates of 50–69 % [2, 3]. Currently, the primary prognostic information for ccRCC is the Fuhrman nuclear grade (a grading system based on nuclear size and morphology) and disease staging at the time of resection [4]. Molecular understanding of this disease has begun to emerge in recent years with two critical papers defining the molecular subtypes of ccRCC [5, 6].

DG is an extracellular matrix receptor which links the extracellular matrix to the actin cytoskeleton [7]. DG is composed of the glycosylated extracellular alpha subunit that is non-covalently bound to the transmembrane beta subunit [8]. DG expression and glycosylation are frequently downregulated in many tumor types [9–17], and loss of αDG glycosylation associates with increased mortality in ccRCC patients [18, 19]. Loss of αDG glycosylation contributes to both invasive and proliferative phenotypes in cancer cells [20–22]. Proper glycosylation is absolutely required for αDG’s function as an extracellular matrix receptor [23]. Therefore derangements of the αDG glycosylation pathway may underlie its dysfunction in cancer. Indeed several studies have identified reduced expression of individual enzymes including LARGE, LARGE2 and β3GNT1 in breast and prostate cancer associated with hypoglycosylation of αDG [12, 21, 22, 24]. However, to date, there has not been a comprehensive analysis of the DG glycosylation pathway in any tumor type and mechanisms underlying loss of αDG glycosylation in ccRCC remain undefined.

The glycosylation of αDG is complex and relies upon the concerted action of at least 13 distinct genes. The initial O-mannosylation of DG requires the combined activity of the protein O-mannosyltransferases 1 and 2 along with the isoprenoid-synthase domain containing protein (ISPD) whose enzymatic activity is unknown [25, 26]. Following O-mannosylation, several enzymes including POMGnT1, fukutin, and fukutin related protein (FKRP) accomplish further glycan modification [27–29] DG requires phosphorylation of its O-mannose for recognition by LARGE and subsequent glycan chain extension [30]. This phosphorylation depends upon a number of more recently identified proteins including glycosyltransferase-like domain containing 2 (GTDC2), β-1,3-*N*-acetylgalactosaminyltransferase2 (B3GALNT2), and SGK196 [31]. From this phosphorylated glycan, LARGE and/or LARGE2, working in concert with B3GnT1, then act through combined xylosyltransferase and glucoronyltransferase activities to generate a repeating disaccharide that is the functional, matrix-binding glycan for DG [21, 30, 32–34]. Loss of function of any of these enzymes, with the exception of LARGE2, has been shown to cause one of a spectrum of muscular dystrophies referred to as the alpha-dystroglycanopathies (for a recent review see [35]). LARGE2, unlike the other glycosyltransferases, is not highly expressed in skeletal muscle or neural tissue, but does show higher-level expression in the kidney [36]. Due to the number of enzymes required for functional DG glycosylation, a large-scale database of tumor genetics is necessary for optimal investigation of the key components of the pathway.

Herein, we utilize the TCGA database to analyze the DG glycosyltransferase pathway and identify a number of genes in this pathway that strongly correlate with tumor grade and stage. We further demonstrate that downregulation of these genes associate with increased overall mortality. Furthermore, we demonstrate a reduction in αDG glycosylation and expression within a case control cohort of ccRCC patients. Finally, we showed that the levels of *GYLTL1B* (the gene encoding the LARGE2 enzyme) mRNA most strongly correlate with hypoglycosylation of αDG in a cohort of ccRCC patient samples*.*

## Materials and methods

### TCGA analysis

All TCGA data was obtained using the University of Iowa Institute for Clinical & Translational Science’s (ICTS) data portal. ICTS created a custom database system for storing the large volumes of data required by the TCGA Dataset. This database utilizes a distributed open source platform, Cassandra from the Apache Foundation. Data was extracted from each of the data files downloaded from TCGA website, then loaded into a representative Cassandra table. Once the data was loaded for each type, we were then able to query and combine the data based on the barcode values for each sample. This combination work has been done in several ways. The first attempt was completed using Perl Scripts and direct access to the files. The current system uses a JAVA Web Application, connecting directly to the Cassandra database via a JDBC Driver (https://research.icts.uiowa.edu/tcga/login.html). Clinical data was obtained from the clinical_kirc.tar.gz (06/14/2012), transcript information from the IlluminaHiSeq_RNASeqV2.Level_3.1.2.0 (01/08/2012), and the methylation status from KIRC, HumanMethylation450.Level_3.6.8.0 (05/13/2013) databases. Only normalized, gene specific transcript data was obtained and integrated (rsem.genes.normalized_results). All gene RNA-Seq by Expectation Maximization (RSEM) values were collected and log-transformed to correct for non-normal distribution. The cBioPortal was utilized for copy number alterations and mutational analysis [37, 38].

### RNA Extraction & qRT-PCR

13 cases of ccRCC resected in the past 12 months and with available tumor and matched normal tissue were selected. One hematoxylin and eosin (H&E) stained slide and 10 unstained slides (6 μm in thickness) were obtained from FFPE tissue blocks. Areas of interest were marked on the H&E stained slides by pathologist. The H&E stained slide was used as a guide for microdissection of tissues from unstained sections. The paraffin flakes were deparaffinized with 1200 μL of xylene, vortexed, and centrifuged (16,000 g x 5 min). The tissue pellet was washed with 95 % ethanol twice before proceeding with RNA extraction. Total RNA extraction was performed with the RNeasy FFPE Kit (Qiagen, Valencia, CA) according to the manufacturer instruction. Reverse transcription (RT) was performed on 1 ng of RNA with iScript cDNA Synthesis Kit (BioRad, Hercules, CA). 1 μl of each RT reaction mixture, TaqMan probes against *GYLTL1B* (Hs00403017_g1)*, DAG1* (Hs00189308_m1)*, LARGE* (Hs00893935_m1)*,* and *ISPD* (Hs00417152_m1) were used with the TaqMan Universal PCR Master Mix) for the subsequent quantitative real-time PCR (qPCR) according to manufacturer’s instruction (Applied Biosystems, Foster City, CA). The results were analyzed by the delta-delta C_t_ method and using the housekeeping gene PPIA (Hs04194521_s1) as a reference for calculation.

### Human samples

All human samples, retrospective and de-identified, were obtained and handled according to the IRB approved protocol #201306718. Formalin-fixed, paraffin-embedded (FFPE) patients’ samples were obtained from the archives of Department of Pathology, University of Iowa (UI) Hospitals and Clinics (Iowa City, IA). All patients had received partial or radical nephrectomy with negative surgical margins. The slides were reviewed and the diagnoses of ccRCC were confirmed by two pathologists. Blocks with the highest tumor percentage and lowest amount of contaminating materials (non-neoplastic cells, necrosis, etc.) were selected for immunohistochemistry and gene expression studies.

### Immunohistochemistry

Immunohistochemistry (IHC) studies for DG were performed by the UI Department of Pathology Core Lab as described previously [22]. Antibodies used for staining include IIH6 (1:100, Santa Cruz Biotechnology, Dallas, TX) and 8D5 (1:100, Leica Biosystems, Buffalo Grove, IL). The pathologists were blinded to staging status at the time of analysis. IHC stained slides were scored by two pathologists independently according to a quartile system whereby: 3: positive (≥90 % of cells showing intensely membrane staining); 2: heterogeneous (regional positivity with >10 % of cells negative); 1: reduced (>10 % of cells negative and decreased intensity of membrane staining); and 0: loss (≤1 % of cells positive). There was 100 % agreement between the 2 independent pathologists. Staining controls are provided as Additional file 1 Figure S1.

### Statistical analysis

To compare expression in tumor-normal matched samples, we carried out paired t-tests of differences in expression on the log scale. Associations between expression and stage/grade were calculated using a proportional odds regression model, adjusting for age and sex. Here, stage and grade were treated as ordinal outcomes. The effects of differential expression on mortality were assessed using a proportional hazards model, again adjusting for age and sex. Separate models were fit for each gene to assess the marginal associations between each gene and disease progression as well as a joint model including expression levels for all genes in order to isolate the effects of individual genes within the context of the entire DG glycosylation pathway. Kaplan-Meier curves were also fit to illustrate the effects of differential expression on overall mortality. Fisher’s exact test was used to assess the association between loss of expression or glycosylation and disease recurrence.

## Results

### The DG glycosylation pathway is perturbed in ccRCC

**Fig. 1:**
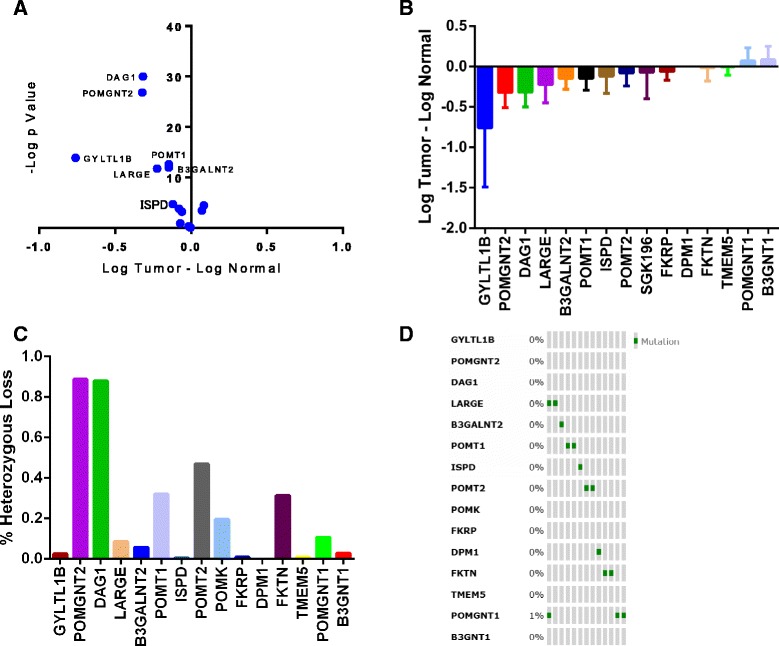
Multiple genes in the DG glycosylation pathway are downregulated during tumor progression. Global analysis of the DG glycosyltransferase pathway describes expression, somatic copy number alterations, and mutational frequency. **a**. Volcano plot demonstrating significance and magnitude change for the analyzed gene set. **b**. Differences in log-transformed expression of the various glycosyltransferases. Error bars represent ± 1 SD. **c**. Utilization of the GISTIC copy number alteration analysis tool through the cBioportal shows frequency of heterozygosity in the analyzed gene set. **d**. Mutational analysis from the cBioportal shows demonstrates the low frequency of mutation within the pathway. Each vertical grouping represents one patient. Mutations are marked in green. Total number of tumors analyzed is 424

We used the TCGA database in order to query the αDG glycosylation pathway to determine which components were most frequently perturbed during tumorigenesis and disease progression. We utilized information from those samples that had matched benign tissue and compared transcript levels of 13 genes known to be involved in αDG glycosylation. In order to visually represent the data, we plotted the findings using both a volcano plot and a relative expression plot to highlight both the magnitude and significance of the changes (Fig. 1a, b). *GYLTL1B*, the gene encoding LARGE2, demonstrated the greatest magnitude change with a nearly 80 % reduction in tumor compared to normal. The two most significantly changed genes were found to be *DAG1*, the gene encoding DG, and *POMGNT2.* Interestingly, these two genes showed nearly identical levels of loss, and when the sample set was analyzed for copy-number variations, we found that *POMGNT2* and *DAG1*, both on chromosome 3p, exhibited nearly 90 % levels of heterozygous loss (Fig. 1c). This strongly suggests that in ccRCC, *POMGNT2* and *DAG1* are co-deleted with the Von Hippel Lindau (VHL) tumor suppressor gene, which also resides on chromosome 3p [39]. Finally, after accounting for these chromosomal alterations, we discovered that LARGE, the homologue of LARGE2, exhibits the next highest level of downregulation with a ~56 % reduction in tumor compared to normal. Importantly, neither *GYLTL1B* nor *LARGE* show a significant loss of heterozygosity. Finally, mutational data was analyzed using Memorial Sloan Kettering’s cBioPortal [37, 38], which revealed that this pathway has a very low mutation rate with the highest frequency being *POMGNT1* at 3/424 tumor samples (Fig. 1d).

### Correlation of DG-associated glycosyltransferases with nuclear grade and tumor stage in ccRCC

**Fig. 2:**
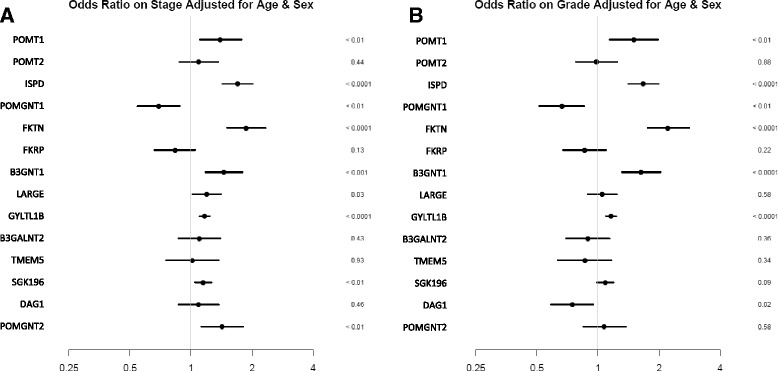
mRNA expression of multiple genes in the DG glycosylation pathway show a significant association with both disease grade and stage. Odds ratios were calculated for the DG glycosyltransferase pathway. **a**–**b**. Stage and grade analysis, adjusted for sex and age, plotted such that a ten-fold downregulation of the indicated genes corresponds to the given increase in odds (right of the axis). 95 % confidence intervals are shown by the horizontal bar. *P* values are listed to the right of the respective genes. Those genes to the right of the y-axis were inversely associated with increasing nuclear grade or tumor stage. For example, a ten-fold down–regulation of ISPD was associated with a 1.7-fold increase in the odds of a higher nuclear grade and stage for the tumor

In order to better assess the association between the expression changes in the DG glycosylation pathway and disease progression, odds ratios were calculated for both Fuhrman nuclear grade and tumor stage of ccRCC (Fig. 2a, b). We found that a number of the genes associated with αDG glycosylation including *POMT1, ISPD, FKTN, B3GNT1, and GYLTL1B,* inversely associated with both grade and stage of the tumor (i.e. decreased expression of these genes was associated with greater odds of higher grade and stage). Interestingly, *POMGNT1* seems to be positively associated with high nuclear grade and tumor stage. Prior work has shown a clear association between DG hypoglycosylation and tumor grade [18]. Our work clearly indicates a similar trend with a number of the glycosyltransferases showing a statistically significant association with both grade and stage. Additionally, we attempted to determine whether any glycosyltransferases associated with lymph node involvement, but low reporting frequency prevented a conclusion to be drawn from this TCGA dataset (data not shown).

### Pathway analysis identifies genes associated with patient mortality

**Fig. 3:**
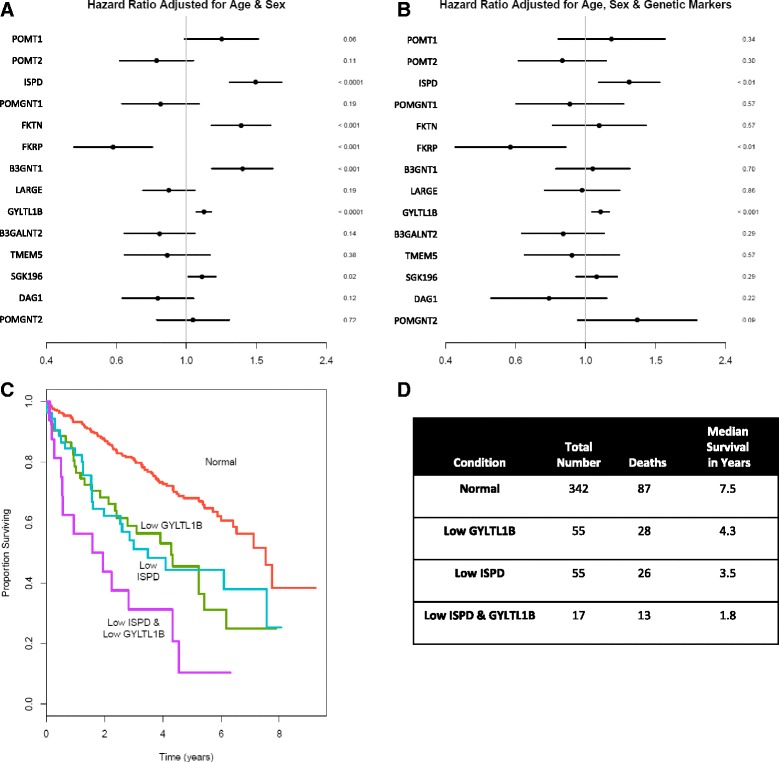
Reduced *GYLTL1B* and *ISPD* expression independently predict increase mortality for clear cell carcinoma patients. Analysis of overall mortality risk, adjusted for sex and age. **a**. Separate analyses for each gene. **b**. A joint analysis of the entire pathway. In both **a** and **b**, a ten-fold downregulation of the indicated genes corresponds to the given increase in risk of death (right of the axis). 95 % confidence intervals are shown by the horizontal bar. P values are listed to the right of the respective genes. **c**. Kaplan-Meier survival curve generated from patients with downregulation of the stated gene greater than 1 standard deviation from the mean. **d**. Table showing number studied, deaths, and median survival values for Kaplan-Meier curve

Due to the associations of glycosyltransferase expression with both grade and stage, we next assessed the possibility that specific genes within this pathway associate with patient survival. We performed survival analysis using a Cox proportional hazards model using these genes and the overall survival data available within TCGA. Initial analysis, corrected for age and sex, revealed an inverse prognostic association with several genes in the DG glycosylation pathway. Downregulation of *ISPD, FKTN, B3GNT1,* and *GYLTL1B,* all of which have been shown to correlate with nuclear grade and tumor stage, were significantly associated with increased patient mortality (Fig. 3a). We next assessed whether any of the genes showed significant correlation since *DAG1* and *POMGNT2* had already been identified as frequently co-regulated, presumably due to their proximity to the VHL gene. We found that a number of the genes showed a statistically significant though moderate correlation (Additional file 2 Figure S2), thus we performed an analysis to adjust for the expression of other genes in the pathway in order to isolate the effect from changes of a single gene. After adjusting for the other genes in the pathway, only *GYLTL1B* and *ISPD* associated with increased mortality (Fig. 3b). This indicates that each of these genes associates independently with mortality and suggests potential roles as drivers of poor clinical outcomes. A Kaplan-Meier survival curve highlights the strong association between loss of expression (one standard deviation below the mean) of both *GYLTL1B* and *ISPD* and survival (Fig. 3c, d). We also see a single gene, *FKRP,* for which upregulation is associated with increased mortality, but the significance of this finding is unclear.

### DG Hypoglycosylation in ccRCC Correlates with Loss of GYLTL1B mRNA

**Fig. 4:**
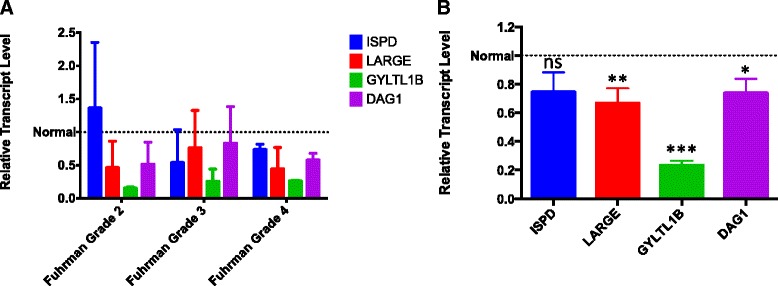
*GYLTL1B* mRNA expression is frequently reduced in ccRCC samples with αDG hypoglycosylation. A. Relative transcript levels normalized to paired controls show no association between expression and Fuhrman nuclear grade. B. Relative transcript values of all paired samples normalized to control. (***, *p* < 0.001; **, *p* = 0.0034; *, *p* = 0.0134; ns, *p* = 0.0746)

In order to determine whether the genes that correlate with increased mortality are responsible for αDG hypoglycosylation in ccRCC, we directly assessed αDG glycosylation (IIH6 staining) and mRNA expression for these genes in recently archived tissues (*n* = 13). All specimens had paired normal renal tissue for comparative analysis. We assessed samples utilizing the glycosylation-sensitive αDG antibody, IIH6, and the βDG antibody, 8D5. This assessment estimates functional glycosylation (IIH6) as well as DG expression (8D5). We found that all 13 samples exhibited complete loss of DG glycosylation (score of 0) and only a moderate loss of DG expression (score of 2) (data not shown). qRT-PCR was performed on these samples to determine the relative expression levels of *DAG1, ISPD, LARGE,* and *GYLTL1B*. Within the TCGA analysis, a reduction in transcript levels was observed in all these genes (Figs. 1 and 3), thus we used them as a targeted subgroup to analyze by qRT-PCR. The samples were assessed by first normalizing to a paired normal tissue to control for inter-patient variability. We first assessed whether any of the four genes in this small sample set correlated with grade, but none showed a significant association (Fig. 4a). However, all four genes exhibited some degree of downregulation with the most significant reduction noted with *GYLTL1B* (Fig. 4a).

### DG Expression and glycosylation are reduced in ccRCC as assessed by immunohistochemical staining

**Fig. 5:**
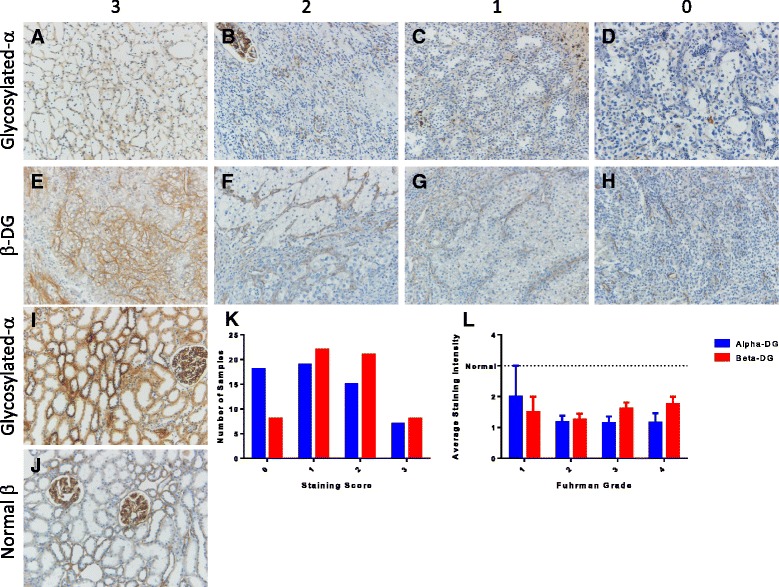
Glycosylated αDG and βDG immunoreactivity is frequently reduced in clear cell renal cell carcinoma. Samples stained for either αDG (IIH6) or βDG (8D5) were scored according to a quartile scoring system whereby 3 = positive (≥90 % of cells intensely positive); 2 = heterogeneous (regional positivity with >10 % of cells negative); 1 = reduced (>10 % of cells negative and decreased intensity of staining); and 0 = loss (≤1 % of cells positive). **a**–**d**. αDG staining and associated scores. **e**–**h**. βDG staining and associated scores. **i**, **j**. Normal tissue demonstrating positive staining. **k**. αDG and βDG staining score distribution. **l**. Scores were grouped by Fuhrman nuclear grade and demonstrate no significant association between staining and grade

Given the findings indicating a potential link between DG glycosyltransferases and patient mortality, we sought to determine whether DG glycosylation could predict disease recurrence following radical nephrectomy. We evaluated 65 patient samples **(**Table 1**)** again utilizing the IIH6 and 8D5 antibodies. Expression of αDG protein was evaluated by IHC staining which was scored as described in Materials and Methods (Fig. 5a-h). As an internal positive control for immunostaining, only cases with adjacent normal tissue showing a score of 3 were included in the study (Fig. 5i, j). Nearly 90 % of samples exhibited staining levels below that of adjacent benign tissue (88 % for αDG and 86 % for βDG). Additionally, αDG exhibited a slightly lower average score (1.186, SEM = 0.131) compared to the average of βDG (1.492,SEM = 0.117). αDG and βDG scores are not correlated (Spearman = 0.1687; *p* = 0.2055) suggesting that a reduction of αDG staining is not secondary to a reduction of the core protein (βDG). As we reported previously for prostate cancer, there are numerous examples in serial sections where αDG staining is lost while βDG staining is retained and we observe similar results in ccRCC (data not shown). Again, no correlation was identified between Fuhrman nuclear grade and the staining of either αDG or βDG (Fig. 5k, l).

**Table 1:** Cohort characteristics of patients utilized within this study

Number of patients	Disease progression	Localized	*P* Value
Age(Range)	57.19 (36.85-74.92)	60.61 (44.38-91.38)	0.1692
Median Time to Relapse (years)	1.59 (0.23-4.91)		
Median Followup (years)	6.47 (4.53-13.84)		
Sex			
Male	21	22	
Female	7	15	0.2898
Smoking Status			
Yes	15	15	
No	13	22	0.3256
Tumor Stage1^1^			
T2	6	15	0.1176
T3	22	22	
Fuhrman Grade			
Grade 1-2	7	22	
Grade 3-4	21	15	*0.0066

Patients treated by radical nephrectomy either remained disease free or experienced recurrence. Chi-square contingency analysis was performed to determine differences between groups. Statistical significance is indicated by *. ^1^Staging is according to TNM classification

**Fig. 6:**
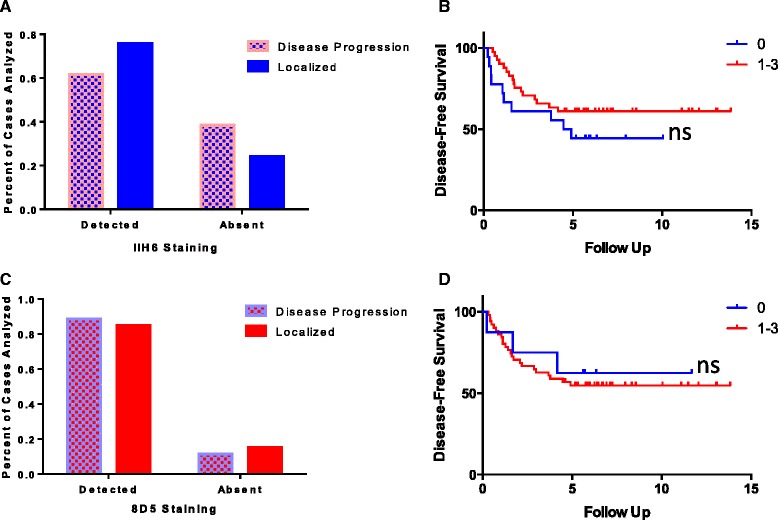
Neither loss of DG glycosylation nor expression assessed by immunohistochemistry associates with disease recurrence. Following scoring, data was compiled and analyzed comparing patients experiencing disease recurrence and those that remained disease-free. Neither αDG (**a**, **b**) nor βDG (**c**, **d**) demonstrated a significant association with disease recurrence when analyzed by Fisher’s exact test (**a**, **c**) or by log-rank assessment (**b**, **d**)

We next assessed whether DG staining could be used as a prognostic biomarker in two patient cohorts that differed in disease recurrence following radical nephrectomy. The two patient cohorts **(**Table 1**)** had largely identical clinical features with the disease progression group exhibiting a slightly higher Fuhrman grade, on average. In order to determine if loss of DG staining was associated with disease recurrence, we compared samples with no DG staining (score of 0) with all other samples (score of 1–3) for both αDG and βDG (Fig. 6). We detected no association between reduced βDG expression and disease recurrence (*p* = 1.00; Fisher’s exact test). Loss of αDG glycosylation was more common in patients with recurrence, although our findings are non-significant (*p* = 0.19), suggesting that a larger cohort may be warranted for future analyses.

### GYLTL1B gene expression associates with increased methylation of its promoter region

**Fig. 7:**
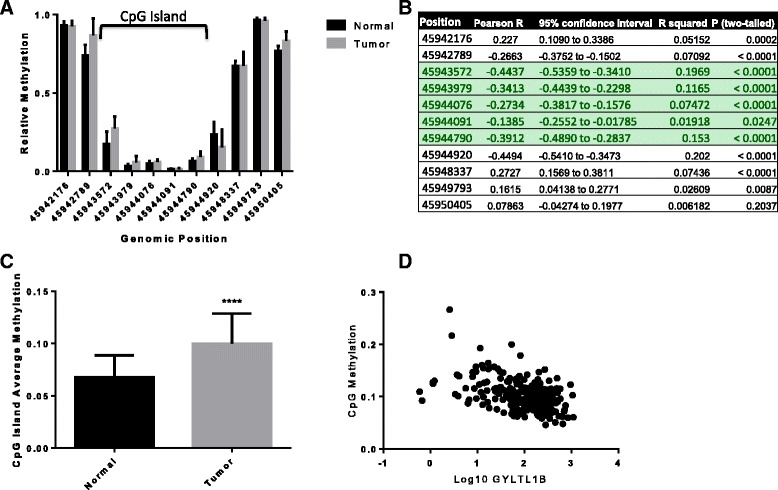
Methylation of the GYLTL1B CpG island negatively correlates with its expression. **a**. Relative methylation analyzed at each of the sites targeted by the Infinium HumanMethylation450 bead array pooled for all samples, normal and tumor, and plotted based upon chromosome position. The CpG island is shown in brackets above. **b. a** table showing the correlation values between methylation and *GYLTL1B* expression. The CpG island is highlighted in green. **c**. All CpG island sites were average per tumor sample and compared to normal controls. **d**. Combined methylation values were plotted against log-transformed *GYLTL1B* RSEM values demonstrating correlation

As LARGE2, the product of *GYLTL1B*, has recently been identified as a critical enzyme for DG glycosylation in prostate cancer, and *GYLTL1B* was the most strongly downregulated gene in ccRCC, we sought to examine the hypothesis that hypermethylation of the GYLTL1B promoter region might contribute to its downregulation utilizing the TCGA database [22]. Inappropriate DNA methylation patterns are frequently observed in various tumor types, and hypermethylation of genes often lead to downregulation of gene expression [40, 41]. We found that the majority of CpG sites demonstrated higher levels of methylation in tumor relative to normal (Fig. 7a). Analysis of the promoter region for *GYLTL1B* using the UCSC genome browser, revealed a CpG island containing 130 CpG sites. We calculated Pearson correlation coefficients for each of the sites, and found that throughout the CpG island, increased methylation was associated with decreased expression of the gene (Fig. 7b). Finally, we analyzed the CpG island in aggregate by averaging the relative methylation of the island for each patient and found that tumor samples demonstrated a statistically significant increase in methylation that exhibited a negative association with gene expression (Fig. 7c, d).

## Discussion

DG has been identified as a potential prognostic biomarker in a number of different malignancies [14–17] and several studies have sought to identify the underlying mechanisms responsible for its hypoglycosylation [12, 21, 22, 24]. Herein we utilized the TCGA database to perform a large-scale, unbiased analysis of the αDG glycosylation pathway. Our results indicate a significant and substantial loss of *GYLTL1B* expression levels through both the TCGA and our tissue-based analysis. LARGE2 was only recently recognized as being a critical mediator of dystroglycan in prostate epithelial cells [22], and these findings support its function in renal epithelium as well. Additionally, this work indicates a strong inverse association between loss of *ISPD* and mortality suggesting a critical role for this enzyme during disease progression. Interestingly, this indicates disruption of dystroglycan glycosylation at both early (ISPD) and late (LARGE2) events involved in the production of the laminin-binding DG glycan. This study is the first to indicate a role for ISPD in contributing to DG hypoglycosylation in tumors. Unlike other genes that have been implicated in DG hypoglycosylation in cancer, such as B3GnT1, LARGE, and LARGE2; ISPD does not yet have a defined glycosyltransferase enzymatic activity and how it is involved in the process of DG glycosylation is unclear [26]. However, we have not shown here that loss of ISPD is causally involved in the loss of DG glycosylation in ccRCC, and this is a clear focus for future studies.

We found that *DAG1* and *POMGNT2* both exhibit high rates of loss of heterozygosity in ccRCC, most likely due to their proximity to the VHL gene. Thus, ccRCC represents a tumor type that is uniquely sensitized to disruption of DG function. Loss of heterozygosity for any of the genes involved in DG glycosylation has not been reported in any other tumor type to date. Despite this, we did not find evidence for secondary mutations in *DAG1* (or in any of the other genes examined). Nonetheless, the frequency of DG disruption at the protein level is very high, with nearly 90 % of α- and β-DG stained samples exhibiting reduction compared to the normal tubules. While this work showed that a number of the DG glycosyltransferases were downregulated in ccRCC tumors, it was still unclear whether downregulation of any of these enzymes lead to hypoglycosylation of αDG. However, our data indicates that *GYLTL1B* was significantly downregulated in ccRCC tumors that showed loss of αDG glycosylation by immunostaining when compared to adjacent normal tissue. Given this observation and our findings from the TCGA dataset, it is highly likely that a reduction of *GYLTL1B* expression is a frequent causative event in αDG hypoglycosylation in ccRCC, consistent with our previous findings in prostate cancer [22].

Building upon the findings above, we sought to determine whether DG could be used as a predictor of disease recurrence following surgical resection with curative intent. While loss of DG immunostaining has previously been linked to grade and increased mortality in patients with ccRCC [18, 19], we did not observe any association between DG expression and disease recurrence in our cohort of 63 patients. We note that these previous studies utilized a binary scoring system of high/low. We believe that our quartile system provides a more accurate representation of the various staining patterns we observed in our studies. Consistent with the previous studies, we did observe a loss of αDG staining in patients that recur, although this finding was not statistically significant in our study. Indeed, a limitation of this aspect of our study is the relatively small size of our retrospective cohort. A larger cohort would be necessary to evaluate significance of the association between DG staining and disease recurrence. It will also be of interest to determine if loss of DG glycosylation is associated with lympho-vascular invasion, which was also not significantly associated with DG glycosylation status (data not shown).

Little is known about the transcriptional control of the genes that are involved in DG glycosylation. We found evidence that the promoter region of *GYLTL1B* is hypermethylated relative to normal tissue indicating that this mechanism may be involved in silencing its expression in ccRCC, similar to what has been proposed for LARGE in breast cancer [24]. While the magnitude of this methylation is not strikingly high, it demonstrates a clear inverse association with gene expression indicating at least a measure of gene regulation. Utilization of bioinformatics allowed us to analyze if any of these genes might be coordinately regulated. This analysis identified a number of genes that have moderate levels of correlation that suggest the possibility of shared upstream regulatory control. (Additional file 2: Figure S2**)**. This finding is interesting in light of our recent publication demonstrating selective regulatory control of LARGE and LARGE2 by SNAIL and/or ZEB1 [42]. Additionally, ZEB1 has been implicated in promoting chromatin modifications, possibly coupled to DNA methylation that could account for LARGE2 promoter hypermethylation [43–45].

## Conclusions

**Fig. 8:**
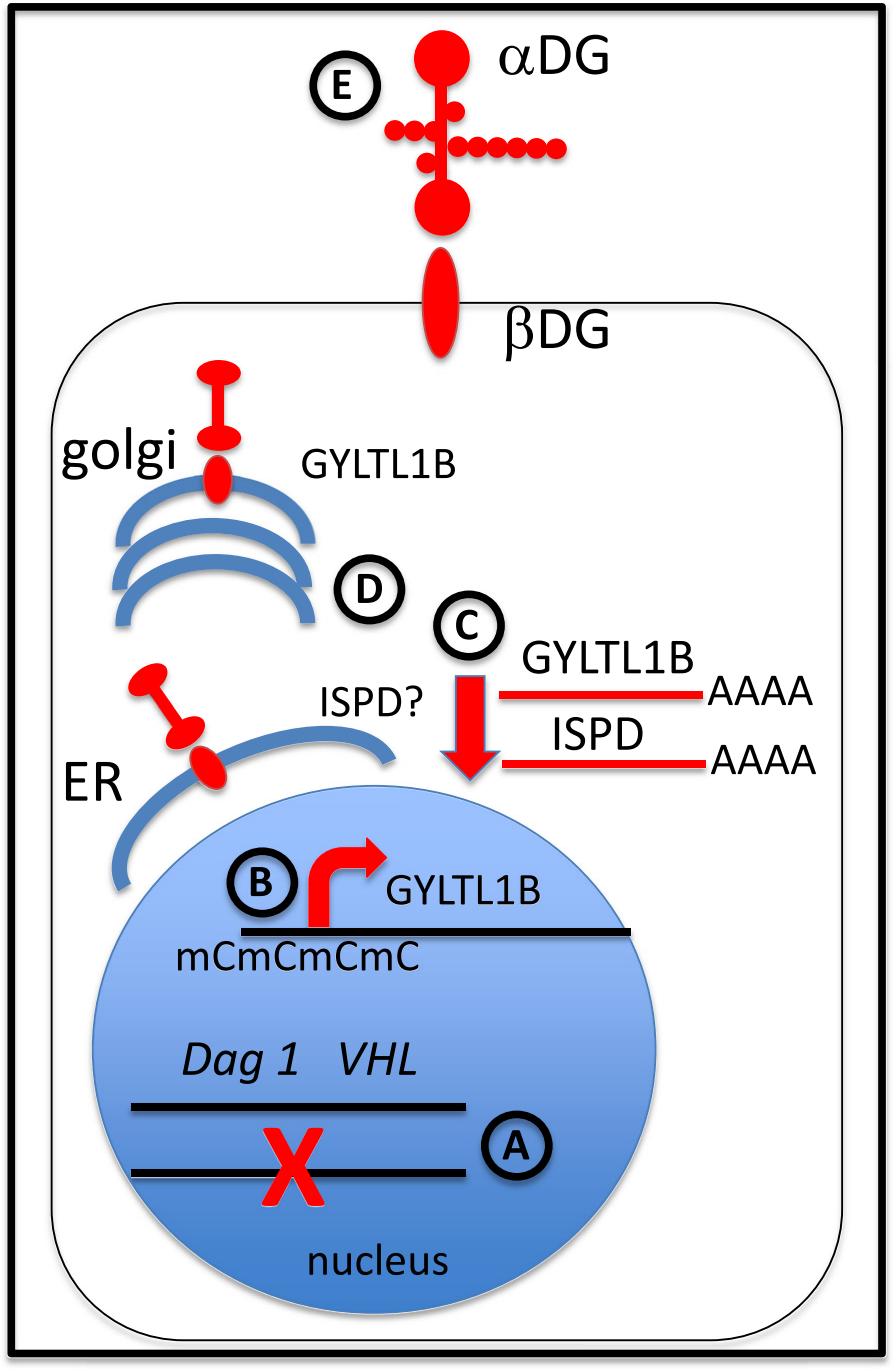
Summary of major findings in this study. See Conclusions for details

Collectively, this work shows that αDG hypoglycosylation is frequent in ccRCC and is often directly associated with reduced expression of *GYLTL1B.* Figure 8 summarizes the major findings in this study related to the expression DG and its glycosylation pathway in ccRCC. (Fig. 8a) The *Dag1* gene is frequently monoallelically lost in ccRCC due to its proximity to the *VHL* gene, however we did not find evidence for mutations in the remaining allele. (Fig. 8b) The GYLTL1B promoter region is frequently hypermethylated. (Fig. 8c) This may contribute to downregulation of GYLTL1B mRNA. ISPD mRNA is also frequently downregulated and both events predict increased mortality for ccRCC patients. (Fig. 8d) Reduced expression of ISPD and GYLTL1B activity in the endoplasmic reticulum (ER) and Golgi, respectively, may result in hypoglycosylation of αDG. Note: the precise enzymatic activity is unknown as is whether it is resident in the ER. (Fig. 8e) Reduced functional glycosylation of αDG on surface of ccRCC cells may reduce their ability to bind extracellular matrix proteins including laminins. While the prognostic implications as assessed by IIH6 immunohistochemistry are limited in this study, the mRNA level analysis in the TCGA dataset illustrates a number of genes involved in αDG glycosylation. These include *GYLTL1B* and *ISPD*, both of which have significant clinical correlations, and may warrant further investigation as prognostic biomarkers.

## Additional files

Additional file 1: Figure S1. Isotype staining controls demonstrate low background signal in staining protocol. Control staining performed on human prostate demonstrates low background staining for both alpha (A) and beta (C) with strong positive signal seen for both (B and D, respectively). (PPTX 5109 kb) Additional file 2: Figure S2. Glycosyltransferase genes show variable levels of correlation across ccRCCC samples. A heat map and associated dendrogram illustrating frequency of correlation between the various genes. Color intensity indicates the strength of the correlation. Blue is negatively correlated and red is positively correlated. (PPTX 141 kb)

## References

[1] Siegel R, Ma J, Zou Z, Jemal A (2014). Cancer statistics, 2014. CA Cancer J Clin.

[2] Cheville JC, Lohse CM, Zincke H, Weaver AL, Blute ML (2003). Comparisons of outcome and prognostic features among histologic subtypes of renal cell carcinoma. Am J Surg Pathol.

[3] Gudbjartsson T, Hardarson S, Petursdottir V, Thoroddsen A, Magnusson J, Einarsson GV (2005). Histological subtyping and nuclear grading of renal cell carcinoma and their implications for survival: a retrospective nation-wide study of 629 patients. Eur Urol.

[4] Sorbellini M, Kattan MW, Snyder ME, Reuter V, Motzer R, Goetzl M, McKiernan J, Russo P (2005). A postoperative prognostic nomogram predicting recurrence for patients with conventional clear cell renal cell carcinoma. J Urol.

[5] Brannon AR, Reddy A, Seiler M, Arreola A, Moore DT, Pruthi RS, Wallen EM, Nielsen ME, Liu H, Nathanson KL (2010). Molecular Stratification of Clear Cell Renal Cell Carcinoma by Consensus Clustering Reveals Distinct Subtypes and Survival Patterns. Genes Cancer.

[6] Cancer Genome Atlas Research N (2013). Comprehensive molecular characterization of clear cell renal cell carcinoma. Nature.

[7] Ervasti JM, Campbell KP (1991). Membrane organization of the dystrophin-glycoprotein complex. Cell.

[8] Ibraghimov-Beskrovnaya O, Ervasti JM, Leveille CJ, Slaughter CA, Sernett SW, Campbell KP (1992). Primary structure of dystrophin-associated glycoproteins linking dystrophin to the extracellular matrix. Nature.

[9] Henry MD, Cohen MB, Campbell KP (2001). Reduced expression of dystroglycan in breast and prostate cancer. Hum Pathol.

[10] Sgambato A, Migaldi M, Montanari M, Camerini A, Brancaccio A, Rossi G, Cangiano R, Losasso C, Capelli G, Trentini GP, Cittadini A (2003). Dystroglycan expression is frequently reduced in human breast and colon cancers and is associated with tumor progression. Am J Pathol.

[11] Sgambato A, Tarquini E, Resci F, De Paola B, Faraglia B, Camerini A, Rettino A, Migaldi M, Cittadini A, Zannoni GF (2006). Aberrant expression of alpha-dystroglycan in cervical and vulvar cancer. Gynecol Oncol.

[12] Akhavan A, Griffith OL, Soroceanu L, Leonoudakis D, Luciani-Torres MG, Daemen A, Gray JW, Muschler JL (2012). Loss of cell-surface laminin anchoring promotes tumor growth and is associated with poor clinical outcomes. Cancer Res.

[13] Parberry-Clark C, Bury JP, Cross SS, Winder SJ (2011). Loss of dystroglycan function in oesophageal cancer. Histopathology.

[14] Jiang X, Rieder S, Giese NA, Friess H, Michalski CW, Kleeff J (2011). Reduced alpha-dystroglycan expression correlates with shortened patient survival in pancreatic cancer. J Surg Res.

[15] Moon YW, Rha SY, Zhang X, Jeung HC, Yang WI, Kwon O, Jeong JH, Cheon SH, Yoo NC, Chung HC (2009). Increments of alpha-dystroglycan expression in liver metastasis correlate with poor survival in gastric cancer. J Surg Oncol.

[16] Coco C, Zannoni GF, Caredda E, Sioletic S, Boninsegna A, Migaldi M, Rizzo G, Bonetti LR, Genovese G, Stigliano E (2012). Increased expression of CD133 and reduced dystroglycan expression are strong predictors of poor outcome in colon cancer patients. J Exp Clin Cancer Res.

[17] Shen JG, Xu CY, Li X, Dong MJ, Jiang ZN, Wang J, Wang LB (2012). Dystroglycan is associated with tumor progression and patient survival in gastric cancer. Pathol Oncol Res.

[18] Sgambato A, Camerini A, Amoroso D, Genovese G, De Luca F, Cecchi M, Migaldi M, Rettino A, Valsuani C, Tartarelli G (2007). Expression of dystroglycan correlates with tumor grade and predicts survival in renal cell carcinoma. Cancer Biol Ther.

[19] Sgambato A, Camerini A, Genovese G, De Luca F, Viacava P, Migaldi M, Boninsegna A, Cecchi M, Sepich CA, Rossi G (2010). Loss of nuclear p27(kip1) and alpha-dystroglycan is a frequent event and is a strong predictor of poor outcome in renal cell carcinoma. Cancer Sci.

[20] Singh J, Itahana Y, Knight-Krajewski S, Kanagawa M, Campbell KP, Bissell MJ, Muschler J (2004). Proteolytic enzymes and altered glycosylation modulate dystroglycan function in carcinoma cells. Cancer Res.

[21] Bao X, Kobayashi M, Hatakeyama S, Angata K, Gullberg D, Nakayama J, Fukuda MN, Fukuda M (2009). Tumor suppressor function of laminin-binding alpha-dystroglycan requires a distinct beta3-N-acetylglucosaminyltransferase. Proc Natl Acad Sci U S A.

[22] Esser AK, Miller MR, Huang Q, Meier MM, Beltran-Valero De Bernabe D, Stipp CS, Campbell KP, Lynch CF, Smith BJ, Cohen MB, Henry MD (2013). Loss of LARGE2 disrupts functional glycosylation of alpha-dystroglycan in prostate cancer. J Biol Chem.

[23] Ervasti JM, Campbell KP (1993). A role for the dystrophin-glycoprotein complex as a transmembrane linker between laminin and actin. J Cell Biol.

[24] de Bernabe DB, Inamori K, Yoshida-Moriguchi T, Weydert CJ, Harper HA, Willer T, Henry MD, Campbell KP (2009). Loss of alpha-dystroglycan laminin binding in epithelium-derived cancers is caused by silencing of LARGE. J Biol Chem.

[25] Manya H, Chiba A, Yoshida A, Wang X, Chiba Y, Jigami Y, Margolis RU, Endo T (2004). Demonstration of mammalian protein O-mannosyltransferase activity: coexpression of POMT1 and POMT2 required for enzymatic activity. Proc Natl Acad Sci U S A.

[26] Willer T, Lee H, Lommel M, Yoshida-Moriguchi T, de Bernabe DB, Venzke D, Cirak S, Schachter H, Vajsar J, Voit T (2012). ISPD loss-of-function mutations disrupt dystroglycan O-mannosylation and cause Walker-Warburg syndrome. Nat Genet.

[27] Chiba A, Matsumura K, Yamada H, Inazu T, Shimizu T, Kusunoki S, Kanazawa I, Kobata A, Endo T (1997). Structures of sialylated O-linked oligosaccharides of bovine peripheral nerve alpha-dystroglycan. The role of a novel O-mannosyl-type oligosaccharide in the binding of alpha-dystroglycan with laminin. J Biol Chem.

[28] Brockington M, Blake DJ, Prandini P, Brown SC, Torelli S, Benson MA, Ponting CP, Estournet B, Romero NB, Mercuri E (2001). Mutations in the fukutin-related protein gene (FKRP) cause a form of congenital muscular dystrophy with secondary laminin alpha2 deficiency and abnormal glycosylation of alpha-dystroglycan. Am J Hum Genet.

[29] Kobayashi K, Nakahori Y, Miyake M, Matsumura K, Kondo-Iida E, Nomura Y, Segawa M, Yoshioka M, Saito K, Osawa M (1998). An ancient retrotransposal insertion causes Fukuyama-type congenital muscular dystrophy. Nature.

[30] Yoshida-Moriguchi T, Yu L, Stalnaker SH, Davis S, Kunz S, Madson M, Oldstone MB, Schachter H, Wells L, Campbell KP (2010). O-mannosyl phosphorylation of alpha-dystroglycan is required for laminin binding. Science.

[31] Yoshida-Moriguchi T, Willer T, Anderson ME, Venzke D, Whyte T, Muntoni F, Lee H, Nelson SF, Yu L, Campbell KP (2013). SGK196 is a glycosylation-specific O-mannose kinase required for dystroglycan function. Science.

[32] Inamori K, Hara Y, Willer T, Anderson ME, Zhu Z, Yoshida-Moriguchi T, Campbell KP (2013). Xylosyl- and glucuronyltransferase functions of LARGE in alpha-dystroglycan modification are conserved in LARGE2. Glycobiology.

[33] Inamori K, Yoshida-Moriguchi T, Hara Y, Anderson ME, Yu L, Campbell KP (2012). Dystroglycan function requires xylosyl- and glucuronyltransferase activities of LARGE. Science.

[34] Hara Y, Kanagawa M, Kunz S, Yoshida-Moriguchi T, Satz JS, Kobayashi YM, Zhu Z, Burden SJ, Oldstone MB, Campbell KP (2011). Like-acetylglucosaminyltransferase (LARGE)-dependent modification of dystroglycan at Thr-317/319 is required for laminin binding and arenavirus infection. Proc Natl Acad Sci U S A.

[35] Wells L (2013). The o-mannosylation pathway: glycosyltransferases and proteins implicated in congenital muscular dystrophy. J Biol Chem.

[36] Grewal PK, McLaughlan JM, Moore CJ, Browning CA, Hewitt JE (2005). Characterization of the LARGE family of putative glycosyltransferases associated with dystroglycanopathies. Glycobiology.

[37] Gao J, Aksoy BA, Dogrusoz U, Dresdner G, Gross B, Sumer SO, et al. Integrative analysis of complex cancer genomics and clinical profiles using the cBioPortal. Sci Signal. 2013;6(pl1). 10.1126/scisignal.2004088 PMC4160307 23550210

[38] Cerami E, Gao J, Dogrusoz U, Gross BE, Sumer SO, Aksoy BA, Jacobsen A, Byrne CJ, Heuer ML, Larsson E (2012). The cBio cancer genomics portal: an open platform for exploring multidimensional cancer genomics data. Cancer Discov.

[39] Gnarra JR, Tory K, Weng Y, Schmidt L, Wei MH, Li H, Latif F, Liu S, Chen F, Duh FM (1994). Mutations of the VHL tumour suppressor gene in renal carcinoma. Nat Genet.

[40] Esteller M, Tortola S, Toyota M, Capella G, Peinado MA, Baylin SB, Herman JG (2000). Hypermethylation-associated inactivation of p14(ARF) is independent of p16(INK4a) methylation and p53 mutational status. Cancer Res.

[41] Eden S, Cedar H (1994). Role of DNA methylation in the regulation of transcription. Curr Opin Genet Dev.

[42] Huang Q, Miller MR, Schappet J, Henry MD. The Glycosyltransferase LARGE2 is Repressed by Snail and ZEB1 in Prostate Cancer. Cancer Biol Ther. 2014. 10.4161/15384047.2014.987078 PMC4623020 25455932

[43] Postigo AA, Dean DC (1999). ZEB represses transcription through interaction with the corepressor CtBP. Proc Natl Acad Sci U S A.

[44] Byles V, Zhu L, Lovaas JD, Chmilewski LK, Wang J, Faller DV, Dai Y (2012). SIRT1 induces EMT by cooperating with EMT transcription factors and enhances prostate cancer cell migration and metastasis. Oncogene.

[45] Sanchez-Tillo E, Lazaro A, Torrent R, Cuatrecasas M, Vaquero EC, Castells A, Engel P, Postigo A (2010). ZEB1 represses E-cadherin and induces an EMT by recruiting the SWI/SNF chromatin-remodeling protein BRG1. Oncogene.

