# Supplementary figures and images for: Downregulation of dystroglycan glycosyltransferases LARGE2 and ISPD associate with increased mortality in clear cell renal cell carcinoma
Source: Mol Cancer. 2015 Jul 30;14:141. doi: 10.1186/s12943-015-0416-z (PMC4518861; doi:10.1186/s12943-015-0416-z)

## Slide 1
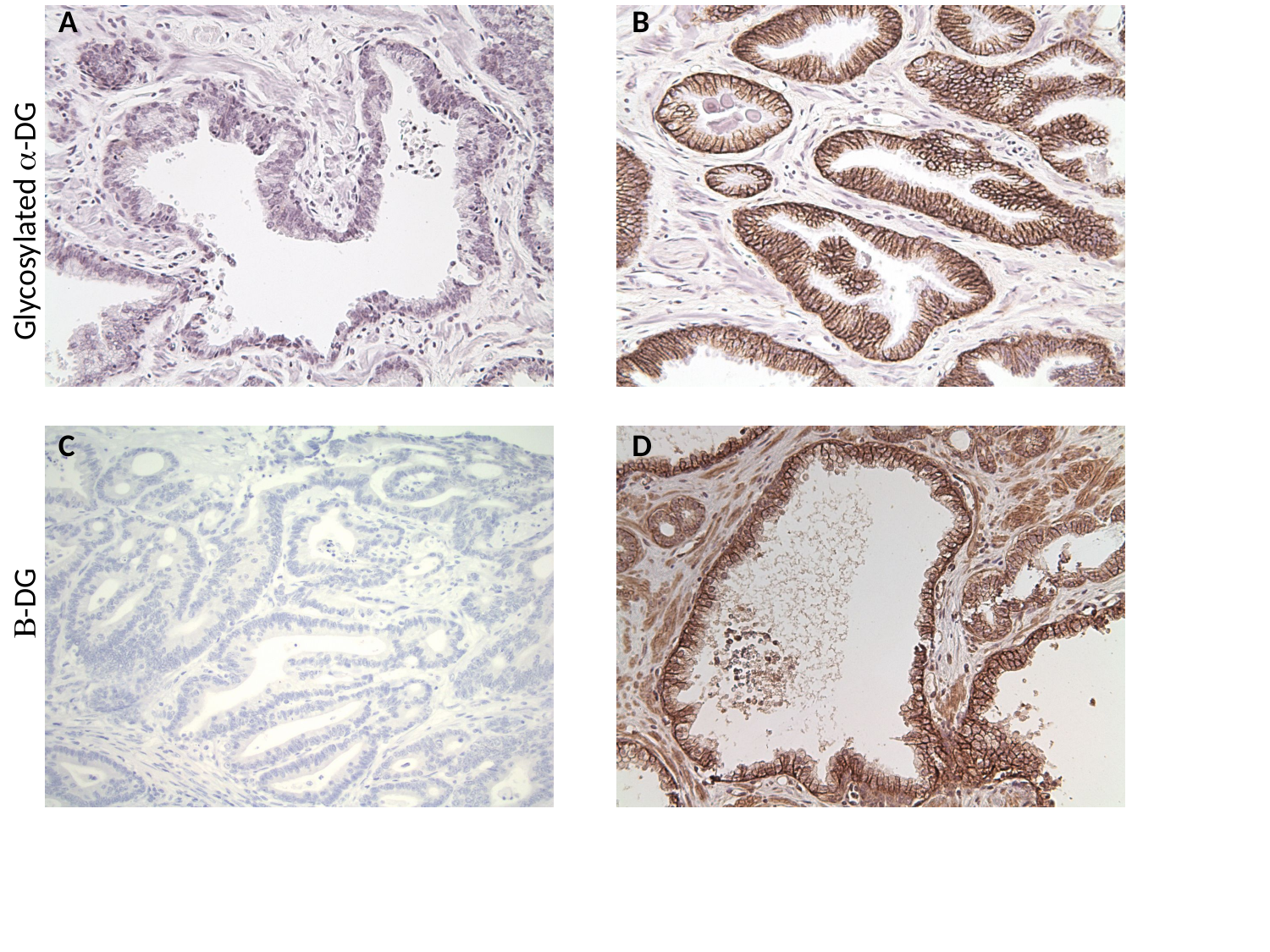

A
B
Glycosylated a-DG
C
D
B-DG

Supplement: Additional file 1: Figure S1. — Isotype staining controls demonstrate low background signal in staining protocol. Control staining performed on human prostate demonstrates low background staining for both alpha (A) and beta (C) with strong positive signal seen for both (B and D, respectively). (PPTX 5109 kb) [file 12943_2015_416_MOESM1_ESM.pptx]
