# Supplementary material for: Downregulation of dystroglycan glycosyltransferases LARGE2 and ISPD associate with increased mortality in clear cell renal cell carcinoma
Source: Mol Cancer. 2015 Jul 30;14:141. doi: 10.1186/s12943-015-0416-z (PMC4518861; doi:10.1186/s12943-015-0416-z)
Supplement: Additional file 2: Figure S2. — Glycosyltransferase genes show variable levels of correlation across ccRCCC samples. A heat map and associated dendrogram illustrating frequency of correlation between the various genes. Color intensity indicates the strength of the correlation. Blue is negatively correlated and red is positively correlated. (PPTX 141 kb) [file 12943_2015_416_MOESM2_ESM.pptx]

## Slide 1
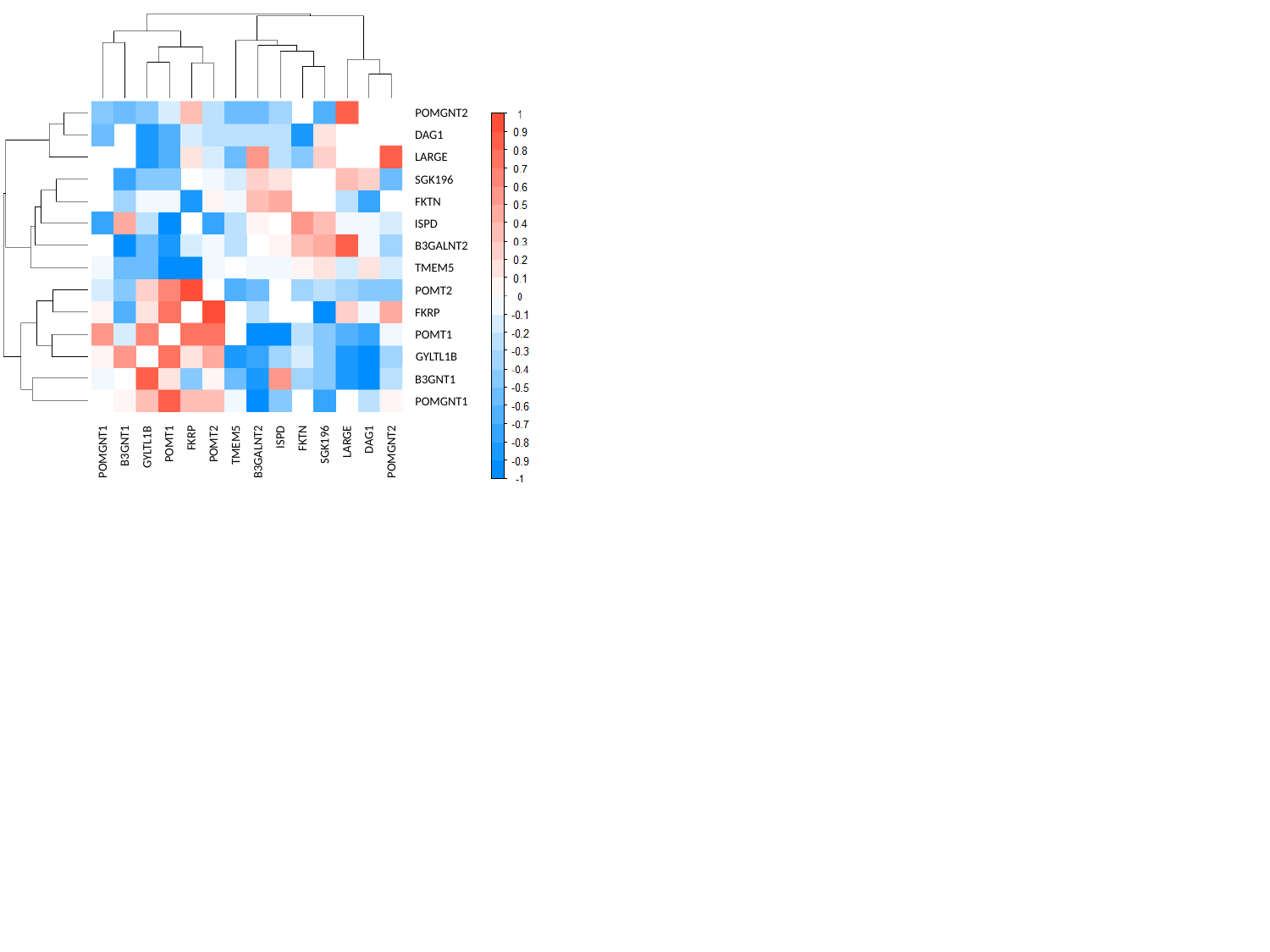

POMGNT2
DAG1
LARGE
SGK196
FKTN
ISPD
B3GALNT2
TMEM5
POMT2
FKRP
POMT1
GYLTL1B
B3GNT1
POMGNT1
ISPD
FKRP
FKTN
DAG1
LARGE
POMT1
POMT2
SGK196
TMEM5
B3GNT1
GYLTL1B
POMGNT1
B3GALNT2
POMGNT2
